# Wartezeiten auf Psychotherapie – Eine differenzierte Analyse der einzelnen Behandlungsphasen

**DOI:** 10.1055/a-2844-7934

**Published:** 2026-05-04

**Authors:** Diethelm Hansen, Max Jacobi

**Affiliations:** 1 Psychologie, MSB Medical School Berlin GmbH, Berlin, Germany; 2 Zentrum für Psychiatrie, Zentrum für Psychiatrie Calw (ZfP) – Klinikum Nordschwarzwald, Calw, Germany

**Keywords:** Wartezeiten Psychotherapie, Sprechstunde, Richtlinienpsychotherapie, psychotherapeutische Versorgung, Behandlungsprozess, Psychotherapy waiting times, initial consultation, Guideline-based psychotherapy, Psychotherapeutic care, psychotherapeutic treatment process

## Abstract

**Einleitung:**

Nach wie vor sind die mittleren Wartezeiten auf den Beginn einer Psychotherapie lang. Wenig untersucht wurde bisher die Differenzierung der Wartezeit auf die einzelnen Phasen des psychotherapeutischen Behandlungsprozesses. Zusätzlich wird die statistische Verzerrungen der Mittelwerte durch Ausreißer wenig berücksichtigt.

**Methodik:**

In einer Befragung von 132 Psychotherapiepraxen in Berlin wurden die Wartezeiten auf die einzelnen Phasen der Psychotherapie ohne Kontakt zu einer Psychotherapiepraxis erhoben. Es wurden die Wartezeiten von Anfrage bis zur 1. Sprechstunde, von der Sprechstunde bis zur 1. probatorischen Sitzung und von letzter probatorischer Sitzung bis Beginn der Richtlinienpsychotherapie erfasst.

**Ergebnisse:**

Alle Daten der Wartezeiten waren nicht normalverteilt. Zwischen Anfrage und 1. Sprechstunde war die Wartezeit im Mittel 4,5 Wochen, im Median nur 2 Wochen, 71% der Praxen gaben Wartezeiten<4 Wochen an. Die Wartezeit zwischen Sprechstunde und 1. probatorischer Sitzung wurde im Mittel mit 5,9 Wochen, im Median ebenfalls mit nur 2 Wochen angegeben, 67% warteten weniger als 4 Wochen. Die Wartezeit zwischen letzter probatorischer Sitzung und Beginn der Richtlinienpsychotherapie war mit einem Mittelwert 2,5 Wochen und einem Median von nur 1 Woche kurz.

**Diskussion:**

Die relevanten Wartezeiten ohne Kontakt zu einer Psychotherapiepraxis von Anfrage bis zur 1. Sprechstunde und von der Sprechstunde auf die 1. probatorische Sitzung sind für viele Patienten deutlich kürzer als die Mittelwerte vermitteln. Allerdings warten zu viele Patient*innen mit mehr als 4 Woche für beide Phasen des Behandlungsprozesses noch zu lange.

## Abstract (en)

**Background:**

Waiting times for the start of psychotherapy remain long on average. However, actual waiting times for the individual phases of the psychotherapeutic treatment process are not usually differentiated. Furthermore, distortions of the average values due to outliers are rarely taken into account.

**Methodology:**

A survey of 132 psychotherapy practices in Berlin was conducted to assess waiting times for the individual phases of psychotherapy for patients who had not yet been in contact with a psychotherapy practice. The waiting times were recorded for the period from initial inquiry to the first consultation session, from the consultation session to the first trial (probationary) session, and from the last trial session to the start of guideline-based psychotherapy.

**Results:**

The data on waiting times were all non-normally distributed. The average waiting time between inquiry and the first consultation was 4.5 weeks, but the median was only 2 weeks, with 71% of practices reporting waiting times of less than 4 weeks. The average waiting time between the consultation and the first trial session was 5.9 weeks, with a median also of just 2 weeks; 67% waited less than 4 weeks. The waiting time between the last trial session and the start of guideline-based psychotherapy was short, with a mean of 2.5 weeks and a median of only 1 week.

**Discussion:**

The relevant waiting times for patients who have not yet contacted a psychotherapy practice – from inquiry to the first consultation and from the consultation to the first trial session – are significantly shorter for many patients than the averages suggest. However, too many patients still wait more than 4 weeks in both of these phases of the treatment process.

## Einleitung

Der Bedarf an Behandlungsangeboten für psychisch erkrankte Menschen hat in den vergangenen Jahren kontinuierlich zugenommen 1 . Zeitnahe Behandlungsangebote sind für diese Patient*innen von besonderer Bedeutung, da gerade bei psychischen Erkrankungen lange Wartezeiten auf eine fachgerechte Behandlung negative Auswirkungen auf den Krankheitsverlauf betroffener Patient*innen haben können 2 . Vor diesem Hintergrund wird unter anderem von der Bundespsychotherapeutenkammer seit Jahren kritisch diskutiert, dass ein zeitnahes psychotherapeutisches Behandlungsangebot häufig nicht sichergestellt werden kann und die Wartezeiten sowohl auf ein Erstgespräch als auch auf den Therapiebeginn deutlich zu lang sind 3 .

U.a. die Psychotherapiereform 2016 (PT-Strukturreform) hatte daher das Ziel, die Wartezeiten auf eine psychotherapeutische Behandlung durch Strukturänderungen zu verkürzen. Als wichtige neue Versorgungselemente wurden die psychotherapeutische Sprechstunde, eine telefonische Erreichbarkeit und die Akuttherapie in die ambulante psychotherapeutische Versorgung eingeführt.

Mehrere Studien haben allerdings gezeigt, dass sich nach der Reform zwar die Wartezeiten auf ein Erstgespräch, also auf die 1. Sprechstunde in einer Psychotherapiepraxis, deutlich verkürzt haben, die Wartezeiten auf eine Richtlinienpsychotherapie allerdings unverändert lang geblieben sind. Dabei wurden mittlere Wartezeiten auf den Beginn der Richtlinienpsychotherapie von 11,5 bis 23,5 Wochen berichtet ( Tab. 1 ), die als zu lang eingeschätzt werden. Bei einer Bewertung dieser Ergebnisse sind mehrere Aspekte zu berücksichtigen:

**Table:** **Tab. 1** Übersicht von Studien zur Wartezeit auf Psychotherapie (in Wochen) und Datengrundlage.

Studie	Erhebungs-zeitraum	Datengrundlage	Erstkontakt – 1. Sprechstunde MW (SD) Median (Min – Max)	1. Sprechstunde – Richtlinienpsychotherapie MW (95% KI) bzw. MW (SD) Median (Min – Max)	Erstkontakt – Richtlinien-psychotherapie MW (SD) bzw. MW (95% KI) Median (Min – Max)
BPtK 2011 4	2011 (vor Reform)	Befragung Psychotherapiepraxen	12,5 (13,6)		17,4 (15,7) alle Praxen 23,4 (16,3) Praxen mit Warteliste
BPTK 2018 5	2017 (nach Reform)	Befragung Psychotherapiepraxen	4,6 (6,6)		19,9 (13,5)
Singer et al. 2021 6	2016 (vor Reform)	Psychotherapiepraxen (Krankenakten)	3,2 (3,9) Median 2 (0, 7)		17,9 (12,6) Median 15 (1,6, 73)
	2018 (nach der Reform)		3,2 (3,9) Median 2 (0, 5,3)		20,1 (1,3) Median 19 (0,6, 104)
Kruse 2024 7	2015 (vor Reform)	Abrechnungsdaten BARMER			11,5 (11,3–11,6)
	2018 (nach Reform)			16,1 (16,1–16,4)	16 (15,8–16,2)
Ritter-Rupp et al. 2023 8	2019–2021	Abrechnungsdaten KV Bayern		19,9 (18,1) Median 13,8 (0–730)

**Abb. 1:**
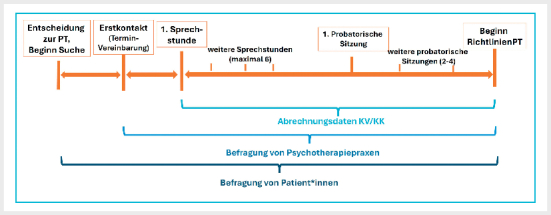
Erfassung der einzelnen Phasen der Wartezeit auf Richtlinienpsychotherapie (RichtlinienPT) differenziert nach den Datenquellen Abrechnungsdaten, Befragung von Psychotherapiepraxen und Befragung von Patient*innen.

1. Bei der Analyse von Wartezeiten kommen unterschiedliche methodische Ansätze zur Anwendung. Neben einer Befragung von Psychotherapiepraxen, der Auswertung von Patientenakten und der Analyse der Abrechnungsdaten der Krankenkassen (KK) bzw. Kassenärztlichen Vereinigungen (KV) können Patient*innen befragt werden. Die jeweiligen Datenquellen erfassen teilweise unterschiedliche Phasen der Wartezeiten ( Abb. 1 ). Die datumsgenauen Abrechnungsdaten der KK bzw. KV zeigen die Dauer zwischen einzelnen abrechnungsrelevanten Terminen in einer oder ggf. auch mehreren Psychotherapiepraxen vom ersten Kontakt in einer Praxis bis zur Durchführung der Psychotherapie. Die Wartezeit vom Erstkontakt in einer Praxis bis zum Erstgespräch hingegen kann nur von Psychotherapiepraxen und von den Patient*innen selbst angegeben werden. Die u.U. längere Suche nach einer Praxis kann nur von den betroffenen Patient*innen berichtet werden. Tab. 1 zeigt eine Übersicht über Studien zur Erfassung der Wartezeit mit Differenzierung nach den unterschiedlichen analysierten Zeitspannen. Tab. 2 zeigt die Wartezeiten aus Sicht der Patient*innen in Patientenbefragungen. Auffällig ist, dass in allen Patientenbefragungen die Wartezeit auf den Therapiebeginn deutlich kürzer angegeben wird im Vergleich zu den Befragungsergebnissen aus Psychotherapiepraxen und den Abrechnungsdaten.
2. In allen dargestellten Studien wurden Mittelwerte und Standardabweichungen zur Beschreibung der Wartezeiten auf die Richtlinienpsychotherapie ermittelt. Nur die Studien von Singer et al. und der Kassenärztlichen Vereinigung (KV) Bayern berichten auch die Mediane sowie Minimum und Maximum. Bei einem Mittelwert der Wartezeit auf den Beginn der Psychotherapie von 19,9 Wochen war der Median in der Analyse der KV Bayern lediglich 13,8 Wochen 8 . Diese Ergebnisse zeigen, dass die Wartezeitdaten rechtsschief verteilt sind und es Ausreißer gibt, die den Mittelwert stark beeinflussen. 50% der Patient*innen in Bayern warteten also weniger als 14 Wochen von der ersten Sprechstunde bis zum Therapiebeginn. Eine Ausreißeranalyse zeigte, dass Patient*innen, die vor Therapiebeginn mehrere Psychotherapiepraxen aufgesucht hatten (20%), eine deutlich längere mediane Wartezeit von 25,4 Wochen hatten als diejenigen, die bei der ersten Psychotherapiepraxis geblieben sind mit 12,1 Wochen. Auch der Vdek hat in einer Analyse der Abrechnungsdaten diese deutlichen Unterschiede zwischen Mittelwert und Median nachgewiesen 12 .
3. Bei allen zitierten Analysen wird als Ende der Wartezeit der Beginn der Richtlinientherapie definiert. Diese Betrachtung lässt außer Acht, dass entsprechend der Psychotherapierichtlinie zwischen dem Erstkontakt und dem Beginn der Leistung „Richtlinientherapie“ mehrere Sitzungen in der Psychotherapiepraxis stattfinden. Neben bis zu 5 weiteren Sprechstunden und diagnostischen Abklärungsterminen finden vor Beginn der Richtlinienpsychotherapie 2–4 probatorische Sitzungen statt 13 . Die Studie der KV Bayern hat ergeben, dass im Durchschnitt 6 Kontakte zwischen 1. Sprechstunde und Richtlinienpsychotherapie stattfinden. Diese Ergebnisse zeigen, dass die vorwiegend betrachtete Zeitspanne von Erstkontakt bis Beginn der Richtlinientherapie keine durchgehende Wartezeit ohne Kontakt zu einer Psychotherapiepraxis ist.

**Table:** **Tab. 2** Übersicht von Patientenbefragungen zur Wartezeit auf Psychotherapie. Zeiträume wurden zur Vergleichbarkeit ggf. zusammengefasst bzw. bei Abweichung der erfassten Zeiträume gesondert gekennzeichnet.

Datenquelle		Bevölkerungsbefragung Kruse et al. 2024 7		BARMER Grobe et al. 2020 9	KBV Kornelius et al. 2019 10	GKV Versicherten-befragung GKV 2022 11
**Erhebungsjahr**		**2012–2017**	**2019**	**2018**	**2019**	**2022**
**Wartezeit** von Anfrage bis Erstgespräch/1. Sprechstunde	<4 Wochen	59%	55%	Nicht erhoben	52%	79% (<31 Tage)
	4 Wochen – 3 Monate	29%	34%		21%	11% (31–60)
	>3 Monate	12%	11%		20%	10% (>60 Tage)
**Wartezeit** von 1. Sprechstunde bis Therapiebeginn	<4 Wochen	83%	76%	66%	74%	94% (<31 Tage)
	4 Wochen – 3 Monate	14%	20%	25%	8%	6% (>30 Tage)
	>3 Monate	3%	4%	9%	5%	

Ergänzend zu dieser globalen Betrachtung der Wartezeit bis zum Therapiebeginn können die differenzierten Wartezeiten während der unterschiedlichen Phasen des Behandlungsprozesses weitere Informationen zur Versorgungssituation liefern.

Zur Konkretisierung der tatsächlichen Wartezeit auf eine psychotherapeutische Behandlung müssen die einzelnen Phasen des gesamten Behandlungsprozesses in Hinblick auf die jeweiligen Zeitintervalle zwischen den einzelnen Terminen in einer Psychotherapiepraxis differenziert betrachtet und analysiert werden. Das wurde bisher lediglich in der Analyse des Vdek 2023 in Teilen untersucht 12 .

Vor diesem Hintergrund haben wir die Hypothese geprüft, dass die tatsächliche Wartezeit, während der keinerlei Kontakt des Patienten zu einer Psychotherapiepraxis stattfindet, für viele Patient*innen kürzer ist, als die häufig als Wartezeit bezeichnete Zeitspanne vom Erstkontakt bis Beginn der Richtlinienpsychotherapie.

## Methoden

### Stichprobe und Datenerfassung

Es wurde eine repräsentative Befragung von psychologischen und ärztlichen Psychotherapiepraxen mit Versorgungsauftrag für Erwachsene in Berlin durchgeführt.

Praxen mit psychotherapeutischem Versorgungsauftrag für Kinder- und Jugendliche sowie Privatpraxen wurden ausgeschlossen. Die anonyme Befragung erfolgte auf der Online-Plattform „SoSciSurf“. Da die Zugangsdaten zu SoSciSurf versendet werden müssen, konnten nur Praxen mit bekannter Email-Adresse kontaktiert werden. Kontaktdaten wurden über die „Arzt- und Psychotherapeutensuche“ der Kassenärztlichen Vereinigung Berlin gesammelt. Zum Zeitpunkt der Befragung wurden in der Bedarfsplanung 2.114 vollzeitäquivalente Vertragsarztsitze für Psychotherapeut*innen geführt. Von 1.834 Praxen wurden Kontaktdaten der Psychotherapeut*innen gehalten, die nicht ausschließlich Kinder und Jugendliche behandeln. Da nicht alle erfassten Psychotherapeut*innen eine Email-Adresse hinterlegt hatten, konnten 873 psychologische Psychotherapeut*innen und 225 ärztliche Psychotherapeut*innen angeschrieben und über die Zielsetzung der Befragung informiert werden.

**Abb. 2:**
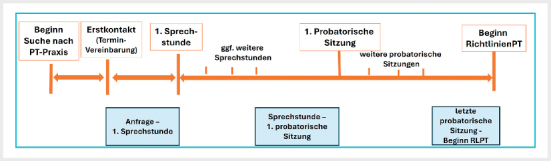
in Psychotherapiepraxen erfassbare echte Wartezeiten zwischen den einzelnen Abschnitten des Behandlungsprozesses.

Es wurden Daten zu Wartezeiten und zur Anzahl angebotener Therapiestunden erhoben. Die Ergebnisse zu den angebotenen Therapiestunden wurden bereits an anderer Stelle publiziert 14 . Der Aufbau des Fragebogens zu den Wartezeiten wurde in drei Teile untergliedert: Erhebung soziodemografischer Daten, Erhebung von Praxisdaten und Erhebung der Zeitintervalle zwischen den einzelnen Behandlungsphasen. Die soziodemografischen Daten beschränkten sich auf das Geschlecht und das Alter der Teilnehmenden. Die Praxisdaten umfassten den Berliner Stadtbezirk, die Praxisorganisation als Einzelpraxis oder Praxisgemeinschaft, die Zulassung als hälftigen oder vollen Versorgungsauftrag sowie das angewendete Richtlinienverfahren. Die Wartezeiten sollten entsprechend folgender definierter Zeitspannen eingeschätzt werden ( Abb. 2 ):

- Erstkontakt bis 1. Sprechstunde
- Sprechstunde bis zur 1. Probatorischen Sitzung
- letzte probatorische Sitzung bis zum Beginn der Richtlinienpsychotherapie

Die Zeiträume von der ersten bis zur letzten Sprechstunde und von der ersten bis zur letzten Probatorischen Sitzung wurden nicht gesondert erhoben, da diese Zeitspannen von der individuell unterschiedlichen Anzahl der jeweiligen Sprechstunden bzw. probatorischen Sitzungen abhängig sind und nicht als Wartezeit angesehen werden können.

### Statistische Analysen

Die Daten wurden mit dem Shapiro-Wilk-Test auf Normalverteilung geprüft. Die Wartezeiten werden als Mittelwert (MW) mit Standardabweichung (SD) sowie als Median mit 25% und 75% Quartil und Minimum und Maximum dargestellt. Ergänzend wurde der Anteil der Praxen ermittelt, die entsprechend den in Tab. 2 zitierten Patientenbefragungen Wartezeiten<4 Wochen, 4–12 Woche oder darüber angegeben haben.

## Ergebnisse

### Stichprobencharakteristika

Die Erhebung wurde von Mai bis Oktober 2024 durchgeführt. Es wurden 1.092 Psychotherapiepraxen einmalig kontaktiert. 301 Praxen haben sich in das Portal eingewählt, 176 haben die Beantwortung begonnen und von 132 konnten vollständige Daten erhoben werden. Das entspricht einer Rücklaufquote von 12%. Es waren Praxen aus allen 12 Berliner Stadtbezirken vertreten. Die Spanne der Rücklaufquoten in den einzelnen Stadtbezirke lag zwischen 8 und 19%. Die Häufigkeit der jeweils eingesetzten Therapieverfahren war in der Stichprobe vergleichbar mit der der kontaktierten Grundgesamtheit ( Tab. 3 ). 92 Praxen hatten einen hälftigen und 40 einen vollen Versorgungsauftrag. Der Anteil der hälftigen Versorgungsverträge war mit 69% identisch wie im gesamten Bundesgebiet 15 . Von allen 132 Praxen konnte die Wartezeit vom Erstkontakt bis zur 1. Sprechstunde, von jeweils 130 Praxen die Wartzeiten von Sprechstunde bis 1. probatorischer Sitzung und von letzter probatorischer Sitzung bis Beginn der Richtlinienpsychotherapie ausgewertet werden.

**Table:** **Tab. 3** Stichprobencharakteristika, Häufigkeit der angewendeten Therapieverfahren in der kontaktierten Grundgesamtheit und der Stichprobe. VT=Verhaltenstherapie, TfP=tiefenpsychologisch fundierte Psychotherapie, AP=analytische Psychotherapie, ST=systemische Therapie. Mehrfachnennungen waren möglich.

	Anzahl Praxen				
	Grundgesamtheit	VT	TfP	AP	ST
**Kontaktiert**	1098	544 (50%)	572 (52%)	251 (23%)	10 (1%)
**Rücklauf**	132	68 (52%)	66 (50%)	30 (23%)	2 (1,5%)

### Wartezeiten ohne Kontakt zur Psychotherapiepraxis

**Abb. 3:**
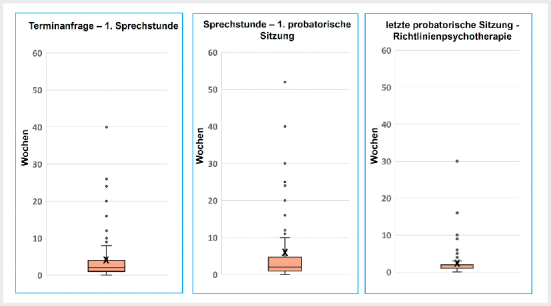
Wartezeiten von Terminanfrage bis 1. Sprechstunde, von Sprechstunde bis 1. probatorische Sitzung und von letzter probatorischer Sitzung bis Richtlinienpsychotherapie. Boxplots, X=Mittelwert.

Die Datenanalyse zeigte für alle Wartezeiten keine Normalverteilung, bei allen 3 Wartzeiten lag eine rechtsschiefe Verteilung der Daten vor. Alle Ergebnisse sind in Tab. 4 zusammengefasst. Abb. 3 zeigt ergänzend die Boxplots und die Mittelwerte für die 3 Wartezeiten. Die teilweise extremen Ausreißer führen dazu, dass bei allen Wartezeiten die Mittelwerte oberhalb oder im Bereich des 75%Quartils liegen.

**Table:** **Tab. 4** Wartezeiten auf jeweils nächste Behandlungsphase. Mittelwert (Standardabweichung), Median mit 25%/75% Quartil und Minimum/Maximum, Anteil der Praxen in Wartezeitkorridoren.

	Wartezeit (Wochen)		
	Erstkontakt – 1. Sprechstunde	Sprechstunde – 1. probatorische Sitzung	Letzte probatorische Sitzung – Beginn Richtlinienpsychotherapie
MW (SD)	4,5 (6,8)	5,9 (9,9)	2,6 (5,2)
Median	2	2	1
25%/75% Quartil	1 / 4	1 / 4	0 / 2
Min/Max	0–40	0–52	0–50
Anteil<4 Wochen	71%	67%	85%
Anteil 4–12 Wochen	20%	20%	13%
Anteil>12 Wochen	9%	13%	2%

Der Mittelwert der Wartezeit vom Erstkontakt bis zur 1. Sprechstunde war 4,5 (SD 6,8) Wochen, der Median 2 Wochen. 71% der Praxen gaben Wartezeiten<4 Wochen auf die 1. Sprechstunde an, in 20% der Praxen mußten die Patient*innen 4 bis 12 Wochen, in 9% länger als 12 Wochen warten.

**Abb. 4:**
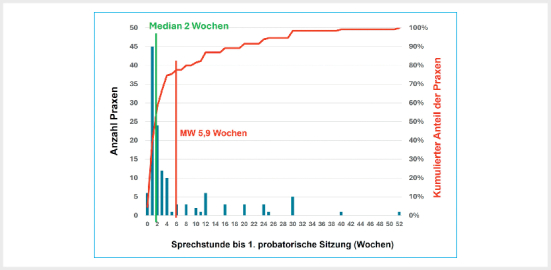
Histogramm und kumulierte Häufigkeit der Wartezeiten zwischen letzter Sprechstunde bis 1. probatorischer Sitzung in 132 Psychotherapiepraxen.

Der Mittelwert der Wartezeit von der Sprechstunde bis zur 1. probatorischen Sitzung war mit 5,9 (SD 9,9) Wochen länger, allerdings lag auch hier der Median bei nur 2 Wochen. 67% der Praxen gaben die Wartezeit bis zur 1. probatorischen Sitzung kürzer als 4 Wochen an, 20% mit 4 bis 12 Wochen und 13% mit länger als 12 Wochen. Die Verteilung der Wartezeiten, die kumulierte relative Häufigkeit der Wartezeit sowie die Diskrepanz zwischen Median und Mittelwert zeigt beispielhaft für diese Wartezeit Abb. 4 .

Die Wartezeit zwischen der letzten probatorischen Sitzung bis zum Beginn der Richtlinienpsychotherapie wurde mit einem Mittelwert von 2,5 (SD 6,2) Wochen angegeben. Der Median lag bei 1 Woche, 85% gaben Wartezeiten kürzer als 4 Wochen an.

## Diskussion

Wir konnten anhand der von uns erhobenen Daten grundsätzlich die statistischen Ergebnisse der KV Bayern sowie der Analyse des Vdek bestätigen, dass Wartezeiten im Rahmen der Psychotherapeutischen Versorgung keine Normalverteilung aufweisen und daher Mittelwerte und Standardabweichungen ein verzerrtes Bild von der tatsächlichen Versorgungssituation geben 8 12 . Die Betrachtung der Mediane, 25% und 75% Quartile sowie die Häufigkeitsverteilung der Wartezeiten erlauben hingegen eine differenzierte Bewertung der Wartezeiten auf eine psychotherapeutische Versorgung.

Die von uns in Berliner Psychotherapiepraxen erhobene Wartezeit zwischen Erstkontakt und 1. Sprechstunde entspricht mit einem Mittelwert von 4,5 Wochen den Ergebnissen anderer Studien ( Tab. 1 ). Der Median von nur 2 Wochen konnte auch von Singer et al. gezeigt werden 16 , 75% hatten in der zitierten Studie nach 5 Wochen einen Sprechstundentermin, in unserer aktuellen Befragung in Berlin gaben sogar 71% der Praxen Wartezeiten<4 Wochen an. Methodisch wurden in der Analyse von Singer et al.

Patientenakten von lediglich 8 Praxen aus den Jahren 2016–2018 ausgewertet. Die geringe Zahl der Praxen könnte die Repräsentativität eingeschränkt haben. Eine weitere Ursache für die kürzeren Wartezeiten in Berliner Praxen 2024 kann die höhere psychotherapeutische Versorgungsdichte in Berlin im Vergleich zum Bundesdurchschnitt sein. In beiden Befragungen der Bundespsychotherapeutenkammer waren die Wartezeiten in Berlin kürzer als in anderen Regionen (5,6).

Interessant ist der Vergleich unserer Daten mit den Ergebnissen der Patientenbefragungen ( Tab. 2 ). In den meisten Patientenbefragungen gaben mit nur 52–59% der Befragten weniger Patient*innen kürze Wartezeiten als 4 Wochen auf den ersten Sprechstundentermin an. Es ist zu vermuten, dass Patient*innen die Wartezeit vom Beginn der Suche nach einer Psychotherapiepraxis an bewerten, also auch die Zeitspanne einbeziehen, die mit der Suche bis zur tatsächlichen Terminvereinbarung vergeht. Wie bereits dargestellt kann diese Zeitspanne weder von den Psychotherapiepraxen noch in den Abrechnungsdaten der Krankenkassen bzw. KV erfasst werden. Die Angaben der Patient*innen dürften die Wartezeiten daher realistischer beschreiben, als es in Praxisbefragungen möglich ist. Insgesamt kann festgestellt werden, dass viele Patient*innen nach vertretbaren Wartezeiten einen Ersttermin bekommen, aber noch zu viele länger als 4 Wochen warten müssen. Die Patientenbefragungen zeigen aber auch, dass die Möglichkeiten der Vermittlung eines zeitnahen Sprechstundentermins in einer Psychotherapiepraxis über die Terminservicestellen der KV noch immer unzureichend genutzt werden 10 .

Die erstmals in Psychotherapiepraxen erfasste Wartezeit zwischen letzter Sprechstunde und 1. probatorischer Sitzung ist entsprechend §12 der Psychotherapierichtlinie die Zeitspanne zwischen der Entscheidung zur weiteren Behandlung und der Einleitung der Richtlinienpsychotherapie 13 . Für viele Patient*innen ist diese Phase eine relevante Wartezeit, da in unserer Studie zwar 67% weniger als 4 Wochen, 33% aber 4 Wochen oder länger auf die 1. probatorische Sitzung warten. In der Analyse des Vdek warteten mit 25% etwas weniger Patient*innen länger als 3 Wochen. Interessant ist der Vergleich unserer Ergebnisse mit den Daten der Patientenbefragungen. Tab. 2 zeigt, dass 66–94% der Befragten die Wartezeit auf den Beginn der Psychotherapie kürzer als 4 Wochen angeben. Diese Quote ist vergleichbar mit den Angaben der von uns befragten Praxen über die Wartezeit von der Sprechstunde bis zur 1. probatorischen Sitzung. Es kann postuliert werden, dass Patient*innen die probatorischen Sitzungen bereits als Psychotherapie wahrnehmen und diese Behandlungsphase nicht als Wartezeit empfinden. Das kann auch die überraschend hohe Zufriedenheit der Patient*innen mit den Wartezeiten in verschiedenen Befragungen erklären 7 . Die kurze Wartezeit zwischen letzter probatorischer Sitzung und Beginn der Richtlinienpsychotherapie scheint hingegen für viele Patient*innen nicht als Problem angesehen zu werden. Diese Wartezeit nach der letzten probatorischen Sitzung ist auch deshalb überraschen kurz, da die Bearbeitungsdauer der Genehmigung der Psychotherapie durch die Krankenkasse zumindest teilweise in diese Zeitspanne fällt.

Die Ergebnisse unserer Befragung von Psychotherapiepraxen zu Wartezeiten auf eine psychotherapeutische Versorgung weisen einige methodische Limitationen auf. Grundsätzlich ist in Frage zu stellen, ob eine Stichprobe von lediglich 12% repräsentativ ist. Aus unserer Sicht kann von einer Repräsentativität unserer Daten ausgegangen werden, da mehrere Stichprobenparameter der Grundgesamtheit entsprechen. Das betrifft sowohl den jeweiligen Anteil der angewendeten unterschiedlichen Therapieverfahren wie auch den Anteil der Praxen mit hälftigem Versorgungsauftrag.

Wie in den meisten Praxis- und Patientenbefragungen wurden die Wartezeiten auch in unserer Studie von den teilnehmenden Praxen geschätzt. Die subjektive Einschätzung der Wartezeiten kann die Ergebnisse daher verzerrt haben. Allerdings scheint dieser mögliche Verzerrungseffekt sehr gering. Wie bereits ausgeführt, weichen die Wartezeiten unserer Befragung nicht oder nur geringfügig von den Wartezeiten aus der Analyse von Patientenakten (16) oder Krankenkassenabrechnungsdaten (12) ab.

Wir haben ausschließlich Psychotherapiepraxen mit Fachkunde für Erwachsene befragt. Unsere Daten geben daher keine Auskunft über die Wartezeiten auf die einzelnen Phasen des Behandlungsprozesses für Kinder und Jugendliche. Unsere Ergebnisse können, wie alle Befragungen von Praxen, keine Auskunft über Wartezeiten von Patient*innen geben, die nach der Entscheidung zur Richtlinienpsychotherapie diese in einer anderen Praxis beginnen. Diese Informationen sind nur in Abrechnungsdaten und Patientenbefragungen erhebbar. Daten der KV Bayern haben gezeigt, dass die Wartezeiten bei diesen Patienten länger sind 8 . Der Anteil der Patient*innen, denen nach den Sprechstunden eine Richtlinienpsychotherapie empfohlen wird, diese dann aber nicht antreten, kann ebenfalls nur in Abrechnungsdaten oder Patientenbefragungen erhoben werden. Daher sollten unsere Ergebnisse durch Analysen von Abrechnungsdaten vertieft werden, die die Wartezeiten zwischen den einzelnen Behandlungsphasen auch bei Patienten mit einem Wechsel zwischen Psychotherapiepraxen einschließen.

## Praxisrelevanz

Zwei Phasen des psychotherapeutischen Behandlungsprozesses sind für die Patient*innen mit relevanten Wartezeiten verbunden: die initiale Wartezeit auf die erste Sprechstunde und die Dauer von der Sprechstunde bis zur ersten probatorischen Sitzung. In 71% bzw. 67% der Praxen sind diese Wartezeiten kürzer als 4 Wochen. Allerdings müssen zu viele Patient*innen 4 Wochen oder länger sowohl auf den ersten Sprechstundentermin als auch auf die 1. probatorische Sitzung nach den Sprechstunden warten.

Zur Analyse und Bewertung der psychotherapeutischen Versorgungssituation sollten nicht nur die Mittelwerte der gesamten Zeitdauer bis zum Beginn der Richtlinienpsychotherapie, sondern auch die echten Wartezeiten zwischen den einzelnen Behandlungsphasen und deren Häufigkeitsverteilung einbezogen werden.
